# Nutrition and Acute Pancreatitis

**DOI:** 10.3390/jcm10040836

**Published:** 2021-02-18

**Authors:** Allison L. Yang

**Affiliations:** Division of Gastroenterology and Hepatology, Weill Cornell Medicine, New York, NY 10065, USA; aly4002@med.cornell.edu

**Keywords:** acute pancreatitis, nutrition, enteral nutrition, parenteral nutrition

## Abstract

Acute pancreatitis (AP) is an acute inflammatory process of the pancreas that is characterized by severe abdominal pain, elevated pancreatic enzymes, and pancreatic changes on abdominal imaging. AP is, by nature, an inflammatory process that leads to protein catabolism and an increased metabolic rate, highlighting the strong need for early nutritional support in the initial management of the disease process. The goal of nutritional support in acute pancreatitis is to correct the negative nitrogen balance to reduce inflammation and improve outcomes. Many trials and multiple systemic reviews and meta-analyses have examined the best modality, timing, and composition of nutritional support for acute pancreatitis. Early enteral nutrition has emerged as an important aspect of the clinical management of AP. This narrative review aimed to provide an overview of the clinical management of nutrition in acute pancreatitis based on the currently available data.

## 1. Introduction

Acute pancreatitis (AP) is an acute inflammatory process of the pancreas. It presents with severe abdominal pain and the diagnosis is supported by elevated pancreatic enzymes and/or characteristic findings on abdominal imaging. The incidence of AP has increased significantly in the past few decades and it is now one of the leading causes of gastrointestinal-related hospitalization in the United States [1,2].

Acute pancreatitis occurs along a clinical spectrum and is categorized as mild, moderately severe, or severe based on the extent of pancreatic injury and the presence and duration of systemic organ failure [3]. While the majority of patients develop mild–moderate AP and recover quickly, up to 20% will develop severe AP with a mortality risk that is estimated to be as high as 30% [4].

The inflammatory nature of AP, which includes the release of inflammatory mediators, leads to systemic inflammatory response syndrome (SIRS), catabolic stress, local pancreatic and peripancreatic necrosis, organ failure, and possible death [5]. The initial management of acute pancreatitis consists of supportive care aimed at targeting the SIRS response with fluid resuscitation, pain control, and nutritional support. The highly inflammatory state leads to the catabolic nature of the disease and puts patients at moderate-to-high nutritional risk [6].

The goal of nutritional support in acute pancreatitis is to prevent malnutrition, correct a negative nitrogen balance, reduce inflammation, and improve outcomes [7]. Numerous ways to provide nutritional support in pancreatitis have been studied, though the topic remains controversial and physician practice patterns are highly variable. Almost 90% of patients are still made “nothing by mouth” or “nil per os (NPO)” at the time of admission based on historical dogma to provide bowel rest, despite more recent evidence supporting the opposite [8].

## 2. How to Provide Nutrition: Enteral versus Parenteral

Traditional principles in the initial management of AP included “bowel rest” to limit the stimulation of exocrine pancreatic secretions [9,10]. Although this may seem preferable in theory, more recent evidence on the gut microbiome and the importance of maintaining the gut–mucosal barrier through enteral nutrition has led to a paradigm shift to strongly favor early enteral nutrition [10]. Current guidelines recommend offering enteral feeding as soon as can be clinically tolerated if medical nutrition therapy is indicated [6,10].

The use of enteral nutrition is well supported by several large-scale meta-analyses. The 2010 meta-analysis by Al-Omran et al. included 8 randomized trials and 348 participants. It clearly showed that early enteral nutrition compared to initial total parenteral nutrition significantly decreased mortality, infection, multi-organ failure, and the need for operative intervention. The subgroup analysis of only patients with severe or predicted severe AP found that mortality was decreased by more than 80% in the enteral nutrition group [11].

These findings have been supported by multiple other meta-analyses. A 2018 meta-analysis by Yao et al. included 5 randomized trials with 348 patients total that also found that compared with parenteral nutrition, enteral nutrition was associated with a significant reduction in overall mortality and the rate of multi-organ failure [12]. Another meta-analysis by Li et al. with 9 randomized controlled trials involving 500 patients showed a significantly lower mortality rate in the enteral nutrition group compared to the parenteral nutrition group, as well as a significantly shorter hospitalization duration. The use of enteral nutrition was also associated with a lower risk of pancreatic infection, organ failure, and the need for surgical intervention [13]. A more recent meta-analysis from Wu et al. in 2018 included 11 randomized trials and a total of 562 patients. They found that enteral nutrition significantly decreased the mortality rate and lowered the risk of infections and other complications compared to parenteral nutrition. The enteral nutrition group had a significantly reduced mean hospitalization time. There were no differences between the groups regarding the development of multiorgan failure [14].

Several more recent single-center studies have also studied this topic and reached the same conclusions. A single-center retrospective study of 171 patients comparing enteral nutrition with parenteral nutrition found a significant reduction in the length of stay, total hospitalization costs, total antibiotic costs, and pain therapy in the enteral nutrition group [15]. A randomized trial by Hui et al. compared enteral nutrition (EN) to total parenteral nutrition (TPN) to enteral plus parenteral nutrition (EN + TPN) and found that both the TPN and EN + TPN groups had significantly increased Ranson scores and Acute Physiology and Chronic Health Evaulation (APACHE II) scores compared to the EN group. The EN group also had significantly lower multiple organ dysfunction and hospital stay lengths [16].

Additionally, even in severe acute pancreatitis, where there is a concern regarding increased intra-abdominal pressure, a small randomized trial of 60 patients showed that early enteral nutrition did not increase intra-abdominal pressure, and might prevent the development of intra-abdominal hypertension [17].

The benefits of enteral nutrition may be in its ability to maintain the gut barrier integrity, thus reducing the translation of bacteria and bacteria-derived endotoxin into the systemic circulation [7]. The use of enteral nutrition may also stimulate intestinal motility and increase splanchnic blood flow [18].

Even in cases of severe acute pancreatitis, where patients may require further interventions, such as percutaneous or endoscopic drainage, and endoscopic or minimally invasive surgical necrosectomy, enteral nutrition and oral food intake can be safe and feasible and guidelines recommend that enteral nutrition can be initiated in the first 24 h after a procedure if the patient is otherwise clinically stable [6].

## 3. When to Start Nutritional Support: Timing of Enteral Nutrition

Current guidelines support early (within 24 h) enteral intake in AP, either by oral refeeding in mild/moderate cases and enteral nutrition support when oral feeding is not tolerated [10]. The majority of patients who have mild-to-moderate AP are able to tolerate oral intake by mouth without issues [6]. Multiple randomized clinical trials have shown that early refeeding (either by mouth or through nutritional enteral support) in these patients leads to a shorter length of stay compared to holding oral intake until after pancreatic enzymes decrease and pain resolves [6].

Early refeeding is supported by a meta-analysis by Horibe et al., which included five randomized controlled trials and found that early oral refeeding significantly decreased the hospital stay lengths. “Early” was defined as either immediately upon admission or patient-directed based upon feelings of hunger. There were no significant differences between early refeeding groups and standard refeeding groups in terms of abdominal pain and distention [19]. Another systematic review comparing early to late enteral feeding (across all routes, including oral and nasoenteral) found early feeding was associated with a reduced length of stay in mild-to-moderate cases and did not increase adverse events across all disease severities [20]. A recent prospective randomized controlled trial in obese patients also suggested that early enteral nutrition prevented obese patients from developing infected pancreatic necrosis, possibly by inhibiting excess inflammation [21].

When oral refeeding is not tolerated, enteral nutritional support should be started within 24 to 72 h of admission [6]. In most moderate-to-severe cases of AP, enteral nutrition can be started early. A recent randomized trial of early nasoenteral feeding versus on-demand oral feeding found that oral feeding can be tried, and if not tolerated, then enteral nutrition can be started within 72 h, even in cases of moderate and severe AP [22]. Further studies on whether early enteral nutrition is beneficial in true severe AP remains to be demonstrated.

Patients with severe AP are more likely to require nutritional support as they are at an increased nutritional risk due to the loss of nutrients, electrolytes, and dysregulation of the acid–base balance [14]. The disease is characterized by protein catabolism, leading to a negative nitrogen balance [23]. The risk of gut barrier dysfunction is also increased, which may allow for the translocation of gut microbiota [24]. Without early nutritional support, this intestinal permeability may increase the risk of infections and ultimately worsen the prognosis of patients with severe AP.

A 2018 meta-analysis by Song et al. included 10 randomized controlled trials and 1051 patients and directly assessed the question of enteral nutrition within 48 h after admission in patients with severe or predicted severe AP. They found that when comparing early enteral nutrition to late enteral nutrition/parenteral nutrition, early enteral feeding was more efficient with respect to reducing mortality, multiple organ failure, need for operative intervention, systemic infection, local septic complications, and gastrointestinal symptoms. There was a trend toward decreased SIRS in the early enteral feeding group, but this was not statistically significant [25].

In contrast to the multitude of studies supporting early (within 24 h) enteral feeding, a 2014 multi-center randomized controlled trial of AP patients at high risk of complications compared early enteral feeding through a nasoenteric tube to an oral diet at 72 h and found no superiority of early nasoenteric tube feeding compared to an oral diet at 72 h regarding reducing the rate of infection or death. In the on-demand oral diet group, one-third of patients ultimately required a nasoenteric tube due to feeding intolerance or mechanical ventilation [22].

## 4. How to Provide Supplemental Enteral Nutrition: Nasogastric versus Nasojejunal

When oral feeding is not tolerated, an alternative approach to nutritional support is needed. Traditionally, a nasojejunal feeding tube was preferred because it allowed for bypassing the inflamed pancreas and resting the organ. However, recent studies have not shown a difference between nasogastric and nasojejunal feeding. The key advantage of nasogastric tubes is that they can often be placed unguided at the bedside, bypassing the need for endoscopy or fluoroscopic guidance.

A 2007 meta-analysis of 3 randomized trials involving 131 patients assessed the effectiveness and safety of early nasogastric enteral nutrition of patients with severe acute pancreatitis. It found no significant differences in mortality in severe AP patients between nasogastric and conventional routes. However, the study was limited since aspiration was not assessed as an outcome [26].

However, two recent meta-analyses concluded that nasogastric feeding as compared to nasojejunal tube feeding is feasible, safe, and tolerated and does not increase complication rates or mortality. The meta-analysis by Chang et al. in 2013 included 3 randomized trials with 157 patients and compared nasogastric versus nasojejunal feeding groups. There were no significant differences seen regarding mortality, aspiration, exacerbation of gastrointestinal symptoms, and the ability to meet caloric needs between the two groups [27]. Likewise, the meta-analysis in 2016 by Zhu et al. included four randomized controlled trials with 237 patients with severe AP and found no significant differences in the incidence of mortality, infection, digestive complications, the achievement of energy balance, or length of hospitalization between the two groups [28]. The 2020 Cochrane review by Dutta et al. included 5 randomized trials with a total of 220 participants and found insufficient evidence to conclude that there was superiority, inferiority, or equivalence between nasogastric and nasojejunal enteral feeding in severe AP [29].

If enteral nutrition support is needed for longer than 30 days, alternative options, such as gastrostomy tubes, should be considered due to the risks and discomfort of prolonged nasoenteric tubes [6]. Data on the long-term outcomes of this approach or in comparison to nasoenteric tubes is limited.

## 5. What to Feed: Composition of Enteral Nutrition

Data on the ideal composition of the ideal diet in AP are limited and success with refeeding has been reported with a wide variety of oral diets, including normal-fat, low-fat, and soft diets of all consistencies [30]. In a randomized trial of 72 patients, refeeding with a full caloric, low-fat diet as soon as bowel sounds were present was safe and well tolerated [31]. In a separate randomized trial of 101 patients with mild acute pancreatitis, no significant difference was observed between patients receiving a soft diet versus a clear liquid diet, and a soft diet led to a decrease in the length of hospitalization [32]. Given these results, guidelines recommend starting a low-fat, soft oral diet when initiating oral feeding in patients with mild AP [6].

When supplemental enteral nutrition is required, guidelines recommend standard polymeric diets given as continuous feeds over cyclic or bolus feeds [6]. A recent 2015 Cochrane review included 15 trials (1376 participants) and found no evidence to support any one specific enteral formula [33]. A 2018 retrospective study out of Japan also suggested no clinical benefit between using an elemental formula compared with a semi-elemental or polymeric formula in patients with AP [34].

## 6. Parenteral Nutrition

While the recommended primary route of nutrition in AP patients should be enteral, certain complications may require the use of parenteral nutrition, including patients with bowel obstructions, abdominal compartment syndrome, prolonged ileus, or mesenteric ischemia [6].

Since severe AP is characterized by significant protein catabolism and an increased metabolic rate with a subsequent negative nitrogen balance, boosting the nitrogen supply is a major goal of nutrition supplementation. The intravenous infusion of amino acids does not affect the pancreatic secretory response [23].

Glucose is the preferred energy source as it can easily be administered and may counteract the gluconeogenesis from protein degradation. It also has the benefit of providing calories while avoiding the use of lipids (especially important in hypertriglyceridemia-related AP). Patients should be monitored closely for hyperglycemia and blood glucose should be corrected with insulin administration as needed [23].

Lipid infusions can be used in pancreatitis as long as hypertriglyceridemia is avoided and can generally be safely used except in cases of hypertriglyceridemia-associated AP [23].

## 7. Supplements

### 7.1. Pancreatic Enzymes

There is no consensus on how to determine the presence of exocrine pancreatic insufficiency in AP. Study data have been limited by small sample sizes.

Only two randomized trials have examined the use of pancreatic enzyme supplementation. In 1995, Patankar et al. evaluated the use of pancreatic enzymes on pain and complication rates in AP in 23 patients. They showed no significant clinical benefits to the use of oral pancreatic enzymes in the initial management of AP [35]. In a double-blind randomized trial in 2014, Kahl et al. showed that 20 out of 56 patients had low fecal elastase values, which is suggestive of pancreatic exocrine insufficiency. Statistical significance was not reached for any of the primary outcomes (recovery from pancreatic exocrine insufficiency) or secondary outcomes (weight, abdominal pain, quality of life). There was a trend toward favoring enzyme use for quality-of-life parameters [36].

### 7.2. Prebiotics and Probiotics

Given the concerns about bacterial translocation across the intestinal barrier leading to infectious complications in AP, numerous trials have evaluated the role of prebiotics and probiotics as a therapeutic adjunct in AP. The findings have been inconsistent and guidelines currently do not support the routine use of probiotics as a supplement to enteral nutrition [6,33]. A 2009 meta-analysis of four studies on the use of probiotics in severe AP showed the probiotics did not reduce the rates of infected necrosis or mortality [37]. A 2014 meta-analysis of 6 trials with 536 patients also assessed the role of probiotics in severe AP. Significant heterogeneity among the trials limited the analysis but the results showed no benefit of probiotics in terms of the infection rate, operation rate, length of hospitalization, or mortality [38]. Additionally, a 2008 randomized, double-blind, placebo-controlled trial involving 298 patients with predicted severe AP showed that probiotic prophylaxis (multispecies preparation) did not reduce the risk of infectious complications and was actually associated with an increased risk of mortality [39].

Earlier, small-scale trials have suggested that prebiotics and probiotics together may have a beneficial role. A small study of 45 patients using *Lactobacillus* and fiber showed a reduction in pancreatic sepsis and the need for surgical interventions [40]. Another study by the same authors in 2007 involving 62 patients using four bioactive fibers and four *Lactobacilli* preparations suggested that synbiotics may lower the risks of multiorgan failure, septic complications, and mortality, though the results were not statistically significant. Larger scale studies are needed to examine this further [41].

### 7.3. Antioxidants

A Cochrane review found very low-quality evidence for antioxidants to support their use as a pharmacologic treatment for patients with AP [42]. Arginine and omega-3 fatty acids have both been studied but are not routinely recommended [7].

However, some studies have suggested that there may be a role for glutamine supplementation in total parenteral nutrition. Glutamine is a nonessential amino acid that has been studied for its antioxidant properties. A 2015 meta-analysis of 11 randomized trials assessed the effect of various antioxidants (5 on glutamine and 6 on other antioxidants) and found that there was a possible benefit of glutamine supplementation in patients with AP in terms of a reduction in hospital stay lengths and a decrease in complications [43]. Another meta-analysis of 12 trials with 505 patients showed that glutamine significantly reduced the risk of mortality and total infectious complications in AP in patients who receive total parental nutrition (i.e., not those on enteral nutrition) [44]. A third meta-analysis of 10 randomized studies with 218 patients showed that compared to controls, glutamine is helpful for elevating albumin levels and decreasing C-reactive protein, infectious complications, and mortality [45].

A recent very small randomized trial assessed the use of enteral glutamine supplementation (18 patients in the treatment arm and 22 patients in the control arm). It found no improvement in infected necrosis or in-hospital mortality with the use of enteral glutamine, but a trend toward improvement in organ function [46].

Given these data, the European Society for Clinical Nutrition and Metabolism recommends parenteral glutamine supplementation for patients on exclusive total parenteral nutrition (not enteral nutrition) [6]. However, the American Society for Parenteral and Enteral Nutrition does not endorse glutamine supplementation in patients with AP [47].

## 8. Pediatric Populations

Data on the optimal method of nutritional support in children with acute pancreatitis is limited. There has only been one randomized controlled trial on early feeding in children, which included 33 patients with a mean age of 11.5. The children were assigned either an immediate, unrestricted diet (early feeding) or initial fasting and intravenous fluids. The median time to starting an oral diet was 19.3 h in the early feeding group versus 34.7 h in the fasting group. There were no differences between the two groups in terms of the time to discharge, serum amylaste/lipase at discharge, or weight change during admission [48]. Additional data to support early patient-directed nutrition in children with acute pancreatitis comes from a study by Ellery et al., which compared 30 prospectively recruited patients with acute pancreatitis (ages 2–21) to retrospective data from 92 patients. The authors found that patient-directed early nutrition was well-tolerated and resulted in a decreased length of hospitalization [49]. These data suggest that similar to adults, early refeeding can be started in children with acute pancreatitis.

## 9. Future Directions

Based on the currently available data, early enteral nutrition is the preferred feeding approach in patients, even those with severe or predicted severe AP. “Early” seems to mean within 24–72 h of admission, and the benefits appear to be a decreased length of stay, decreased complication rates, improved mortality, and increased cost-effectiveness.

Despite the multitude of systematic reviews and meta-analyses on the modality, timing, and constitution of nutritional supplementation in acute pancreatitis, the results are often heterogeneous, as trials study various time frames (some consider 24 h, some consider 48 h, some consider 72 h). The data are not always comparable. Open questions also remain regarding the best modality of medical nutrition therapy in patients undergoing interventions, such as endoscopic/surgical drainage or necrosectomy, as well as long-term nutrition in severe AP requiring the use of percutaneous gastrostomy tubes. Larger, randomized trials will be needed to sufficiently answer these controversies. 

## Data Availability

Not applicable.
